# Dengue type 4 in Rio de Janeiro, Brazil: case characterization following its introduction in an endemic region

**DOI:** 10.1186/s12879-017-2488-4

**Published:** 2017-06-09

**Authors:** Manoela Heringer, Thiara Manuele A Souza, Monique da Rocha Q Lima, Priscila Conrado G Nunes, Nieli Rodrigues da C Faria, Fernanda de Bruycker-Nogueira, Thaís Chouin-Carneiro, Rita Maria R Nogueira, Flavia Barreto dos Santos

**Affiliations:** 1 Viral Immunology Laboratory, Oswaldo Cruz Institute/FIOCRUZ, Av Brasil 4365, Manguinhos, Rio de Janeiro, RJ 21045-360 Brazil; 2 Flavivirus Laboratory, Oswaldo Cruz Institute, Rio de Janeiro, Brazil

**Keywords:** Dengue virus type 4, Laboratorial diagnosis, Phylogeny, Endemic, Rio de Janeiro, Brazil

## Abstract

**Background:**

Due to the populations’ susceptibility, DENV-4 introduction in 2010 led to the occurrence of explosive epidemics in the following years in Brazil. In 2011, DENV-4 was identified in Rio de Janeiro (RJ) and it was prevalent in 2012 and 2013. Here, we aimed to characterize clinical, epidemiological and laboratorial aspects of DENV-4 cases after this serotype introduction in an endemic scenario.

**Methods:**

Dengue suspected cases (*n =* 3727) were received and analyzed from January 2011 to December 2013, during outbreaks occurred in RJ, Brazil. Samples were submitted to virological, serological and molecular methods for case confirmation. DENV-4 cases (*n =* 705) were characterized according to the type of infection, disease severity and, viremia levels and NS1 antigenemia were accessed. Representative strains were partial sequenced for genotyping.

**Results:**

DENV-4 was identified in 44.2% (705/1593) of dengue positive cases, virus isolated in 48.7% of the cases. Anti-DENV IgM was detected in 39.4% of the cases, however an increased detection was observed in cases with ≥4 days of symptoms (57.0%). NS1 antigen was identified in 41.5% of DENV-4 cases however, after immune complexes dissociation, the detection significantly increased (87.6%). Females were more affected than males, so did children aged 11–15 years old. Primary cases were more frequently observed than secondary ones and most of them were classified as dengue. No differences on NS1 antigenemia and viraemia within the groups were observed. Despite the higher frequency of severe disease on individuals >65 years old, no differences were observed among the groups and type of infection. However, DENV-4 fatal cases were more frequent on secondary infections (57.1%). DENV-4 Genotype II was identified with a probable origin from Venezuela and Colombia.

**Conclusions:**

It has been shown that laboratorial diagnosis is still a reliable tool for the disease surveillance, detecting and confirming emerging epidemics. Despite the occurrence of secondary infections, most DENV-4 cases presented a mild disease. As RJ is endemic for dengue, high rates of secondary infections would be expected. Despite the existence of two genotypes, only Genotype II was identified in our study.

## Background

Dengue is one of the most important arboviruses in the world and the World Health Organization (WHO) estimates that between 70 and 500 million people are annually infected worldwide [1]. Dengue viruses (DENV 1–4) belong to the family *Flaviviridae*, genus *Flavivirus* [2] and based on the analysis of the envelope gene sequences [3, 4] or complete sequencing of the viral genome [5], four genotypes are characterized for DENV-4: Genotype I represented by strains from Thailand, Philippines, Sri Lanka and Japan (from Southeast Asia); Genotype II represented by strains from Indonesia, Malaysia, Tahiti, the Caribbean and the Americas; Genotype III represented by recent strains in Thailand that were different from the others; and Genotype IV represented by wild strains of Malaysia [3–5].

After 63 years without reported cases in Brazil, reinfestation by vector *Aedes aegypti* in the 70’s led to epidemics in Boa Vista, Roraima in 1981–1982 [6]. However, dengue became a public health problem in the country when the DENV-1 was identified in the serum of patients in an epidemic in the state of Rio de Janeiro (RJ) in 1986 [7]. The introduction of DENV-2 in 1990, also in the state of RJ [8], led to an increase in the disease severity and the first dengue hemorrhagic fever (DHF) cases were reported in the country [9]. DENV-3 introduction in RJ and in the country in 2000 caused one of the most severe epidemics reported in the country in 2002 [10, 11]. In 2007–2008, the country experienced the most severe epidemic in terms of morbidity and mortality, in addition to severe cases in children due to the DENV-2 re-emergence. RJ alone was responsible for 255,818 cases [12–14]. In 2009, DENV-1 re-emerged in the southeast region of the country and it was the serotype detected in 50.4% of the viral isolations, displacing DENV-2 and DENV-3 [15]. In July of 2010, DENV-4 was isolated in Roraima [16], 28 years after its first detection in that same State and soon this serotype spread other States, including RJ [17]. Despite the epidemic caused by DENV-1, DENV-4 could be isolated during the disease surveillance supported by the performed laboratorial diagnosis. Due to the populations’ susceptibility to this newly introduced serotype, explosive epidemics in the country were a real threat. Furthermore, despite being known as a mild serotype, the impact of the emergence of DENV-4 in an endemic region where the other three serotypes were circulating was unknown. This study aimed to analyze the epidemiology of DENV-4 cases occurring in Rio de Janeiro after this serotype introduction, the role of the different laboratory methodologies for case confirmation, the association of viremia and NS1 antigenemia with disease severity and the surveillance of the circulating genotype.

## Methods

### Dengue suspected cases

Dengue suspected cases were received during a surveillance program performed by the Flavivirus Laboratory, Oswaldo Cruz Institute, FIOCRUZ, Regional Reference Laboratory for the Brazilian Ministry of Health, located in RJ, from January 2011 to December 2013. Acute serum samples (up to the 7th day after the onset of the symptoms) stored at −70 °C were submitted for virus isolation, RT-PCR and NS1 antigen capture ELISA. Convalescent samples (> the 7th day after the onset of the symptoms) stored at −20 °C were tested for MAC-ELISA and IgG-ELISA tests.

### Dengue cases classification

Dengue confirmed cases were classified according to the 2009 WHO classification [18] and grouped as follows: dengue without warning signs: patients living in and/or traveling to dengue endemic area, presenting fever and two of the following symptoms: nausea, vomiting, rash, aches, pain, positive tourniquet test and leukopenia; dengue with warning signs (DWAS): dengue patients with any of the following warning signs: abdominal pain or tenderness, persistent vomiting, clinical fluid accumulation, mucosal bleeding, lethargy or restlessness, liver enlargement >2 cm, and an increase in hematocrit concurrent with rapid decrease in platelet count and severe dengue (SD): “dengue patients presenting at least one of the following:” severe plasma leakage (leading to shock and fluid accumulation with respiratory distress), severe bleeding evaluated by clinicians, severe involvement of liver by aspartate aminotransferase (AST) or alanine transferase (ALT) >1000 U, central nervous system with impaired consciousness, and severe involvement of the heart and other organs.

### Virus isolation

Virus isolation was performed by inoculation into C6/36 *Aedes albopictus* cell line [19] and isolates were identified by indirect fluorescent antibody test (IFAT) using serotype-specific monoclonal antibodies [20].

### Immunoglobulin M (IgM) antibody capture ELISA (MAC-ELISA)

The Panbio dengue IgM Capture ELISA (E-DEN01M, Brisbane, Australia) was used for the qualitative detection of anti-DENV IgM antibodies in serum for case confirmation according to the manufacturer’s instructions.

### Immunoglobulin G (IgG) antibody detection ELISA (IgG-ELISA)

The IgG-ELISA previously described [21] was performed for the characterization of dengue immune response as primary or secondary infections, in dengue cases previously confirmed by virus isolation, RT-PCR and/or MAC-ELISA.

### Viral RNA extraction

Viral RNA was extracted from serum using QIAamp Viral RNA Mini kit (Qiagen, Hilden, Germany) following the manufacturer’s instructions and stored at −70 °C for DENV typing.

### Reverse transcriptase-polymerase chain reaction (RT-PCR)

Conventional RT-PCR was performed as described by Lanciotti et al. [22] for DENV detection and typing from acute phase samples.

### RT-PCR amplification and sequencing of the gene E

For genotyping and phylogenetic analysis, representative samples were selected from the 3 years of epidemic and from distinct regions within the state of Rio de Janeiro. Those DENV-4 isolates (*n =* 12) were partially sequenced (C/prM/M/E genes), Table 1. Five microliters of the extracted RNA was reverse transcribed into cDNA and amplified for sequencing using AccessQuick™ RT-PCR System (Promega Corporation, Wisconsin, USA). The thermocycling parameters consisted of one cycle of reverse transcription (42 C/60 min), followed by 40 cycles of denaturation (94 C/35 s), annealing (59 C/1 min) and extension (72 C/2 min), ending with a final extension cycle (72 C/10 min), in a GeneAmpPCR System 9700 (Applied Biosystems, California, USA). The cDNA fragments amplified by RT-PCR were directly sequenced in both directions using the BigDye Terminator Cycle Sequencing Ready Reaction Kit (Applied Biosystems, Foster City, CA) version 3.1 and analyzed in an automatic sequencer 3100 (Applied Biosystems, Foster City, CA). Alignments were performed using CLUSTALW2 (http://www.ebi.ac.uk/Tools/msa/clustalw2/). Phylogenetic trees were constructed using the MEGA 7 (http://www.megasoftware.net/), by the “Neighbor-joining” method, according to the Tamura-Nei 93 model and Gamma distribution (TN93 + G), with a bootstrap of 1000 replications.

**Table 1:** DENV-4 strains (*n =* 12) isolated in Rio de Janeiro, from 2011 to 2013 used in this study for partial genome sequencing and genotyping

ID/state/year of isolation	Origin of strain (cell line)	Case classification (WHO, 2009)	Genbank accession number
487_11/RJ/2011	Isolated (C6/36)	Dengue	KY084510
6338_11/RJ/2011	Isolated (C6/36)	Dengue	KY084511
1128_12/RJ/2012	Isolated (C6/36)	Dengue	KY084512
3178_12/RJ/2012	Isolated (C6/36)	Dengue	KY084513
3603_12/RJ/2012	Isolated (C6/36)	Dengue	KY084514
5340_12/RJ/2012	Isolated (C6/36)	Dengue	KY084515
408_13/RJ/2013	Isolated (C6/36)	Dengue	KY084516
2663_13/RJ/2013	Isolated (C6/36)	Dengue	KY084517
2848_13/RJ/2013	Isolated (C6/36)	Dengue	KY084518
2960_13/RJ/2013	Isolated (C6/36)	Severe Dengue (Fatal)	KY084519
3248_13/RJ/2013	Isolated (C6/36)	Dengue	KY084520
3602_13/RJ/2013	Isolated (C6/36)	Dengue	KY084521

*ID* Identification, *RJ* Rio de Janeiro, *WHO* World Health Organization

### Real time RT-PCR assay for viremia quantitation

For the viremia quantification of DENV-4 cases (*n =* 16 [8 dengue and 8 severe dengue]), the TaqMan assay was performed as described previously by Johnson [23]. Due to the small number of severe cases, viremia was randomly analyzed only in 8 samples of each group, for comparison purposes.

### NS1 antigen capture ELISA

For the NS1 antigen capture, the Platelia™ Dengue NS1 Ag-ELISA kit (Biorad Laboratories, Marnes-La-Coquette, France) was used according to the manufacturer’s protocol. All samples were tested between 1 and 12 days after disease onset. For a better sensitivity for the NS1 capture test, an antigen-antibody complex dissociation protocol performed as previously described by our group [24]. Briefly, the heat-mediated dissociation method was used by boiling 50 μL of serum at 100^0^ C in a water bath for five minutes.

The NS1 antigenemia quantification was performed using a standard synthetic antigen curve, based on an equation (y = 1,321× + 0.1271) with R^2^ = 0.9542. The standard curve was established using synthetic NS1 protein based on the DENV-4 Dominica 814,669/1981 strain (Native Antigen Company, Oxforshire, United Kingdom) with a 10 fold-dilution. All samples were tested between 1 and 12 days after disease onset.

### Statistical analysis

The chi-square and/or Fisher exact tests were used to assess the significance between categorical variables by using Epi Info 7.0.9.34 (Center for Disease Control and Prevention, Atlanta).

## Results

A total of 3727 dengue suspected cases from the state of RJ was analyzed from January 2011 to December 2013. After the confirmatory laboratorial diagnosis, 1593 cases were confirmed as dengue positive, and 705 (44.2%) due to DENV-4 infections. Not all analysis, such as those by gender, age, immune response and disease severity were performed in all 705 DENV-4 due to the lack of information in some cases.

The highest percentage of the cases received and confirmed in this study were from the Metropolitan Region of RJ (89.9%; 634/705), and the most affected municipalities by DENV-4 in 2011–2013 were Niterói and Rio de Janeiro, followed by Campos dos Goytacazes, located in the North region of the state, where 44.9% (317/705), 36.8% (260/705) and 7.5% (53/705) of infections by this serotype were confirmed, respectively.

The RT-PCR failed to identified DENV-4 in only one sample (Table 2), which was confirmed by virus isolation, that identified the serotype in 48.7% (298/612) of the samples tested. The detection of anti-DENV IgM, considering both acute and convalescent samples, was possible in 39.4% (104/264) of the cases. However, considering cases with ≥4 days of illness, this marker was detected in 57.0% of the cases. DENV NS1 antigen was identified in 41.5% (231/557) of the DENV-4 cases by using the NS1 antigen capture ELISA according to the manufacturer’s instructions, however, after the addition of an antigen-antibody complex dissociation step, the assay sensitivity significantly increased and confirmed 87.6% (488/557; *p = 0.001*) of the samples tested, Table 2.

**Table 2:** Distribution of DENV-4 cases analyzed in the State of Rio de Janeiro, Brazil, from 2011 to 2013 and confirmed according to the routine laboratorial diagnosis methodologies used

Methodology/Year	Positive cases/Total analyzed (%)	*p* value
Virus isolation	298/612 (48.7)	
RT-PCR	704/705 (99.8)	
MAC-ELISA	104/264 (39.4)	
NS1 Capture ELISA	231/557 (41.5)	0.001
NS1 Capture ELISA after dissociation	488/557 (87.6)	

**Fig. 1:**
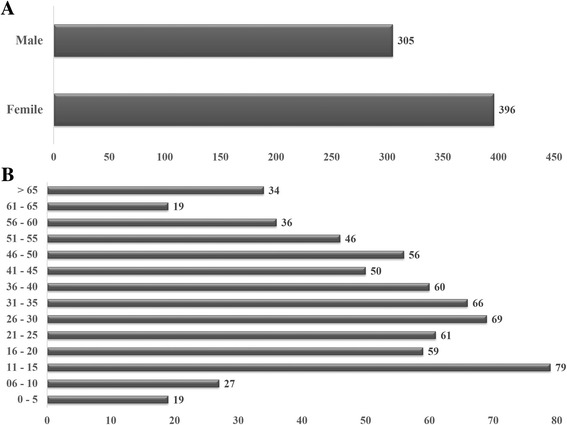
Distribution of DENV-4 cases by gender (**a**) and age group (**b**), during the epidemic occurred in Rio de Janeiro, Brazil from 2011 to 2013

DENV-4 cases were more frequently observed in females (*n =* 396) than males (*n =* 305), and a higher number of cases were observed on children aged 11–15 year old, and was no significant differences were observed between age groups (Fig. 1).

**Fig. 2:**
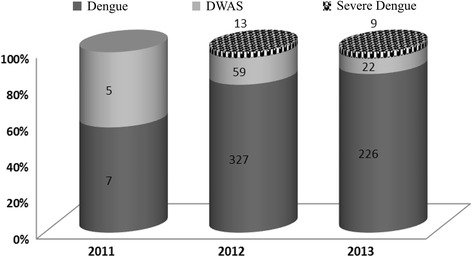
Yearly distribution of dengue, dengue with warning signs (DWAS) and severe dengue cases by year of occurrence, Rio de Janeiro, Brazil, 2011 to 2013

**Fig. 3:**
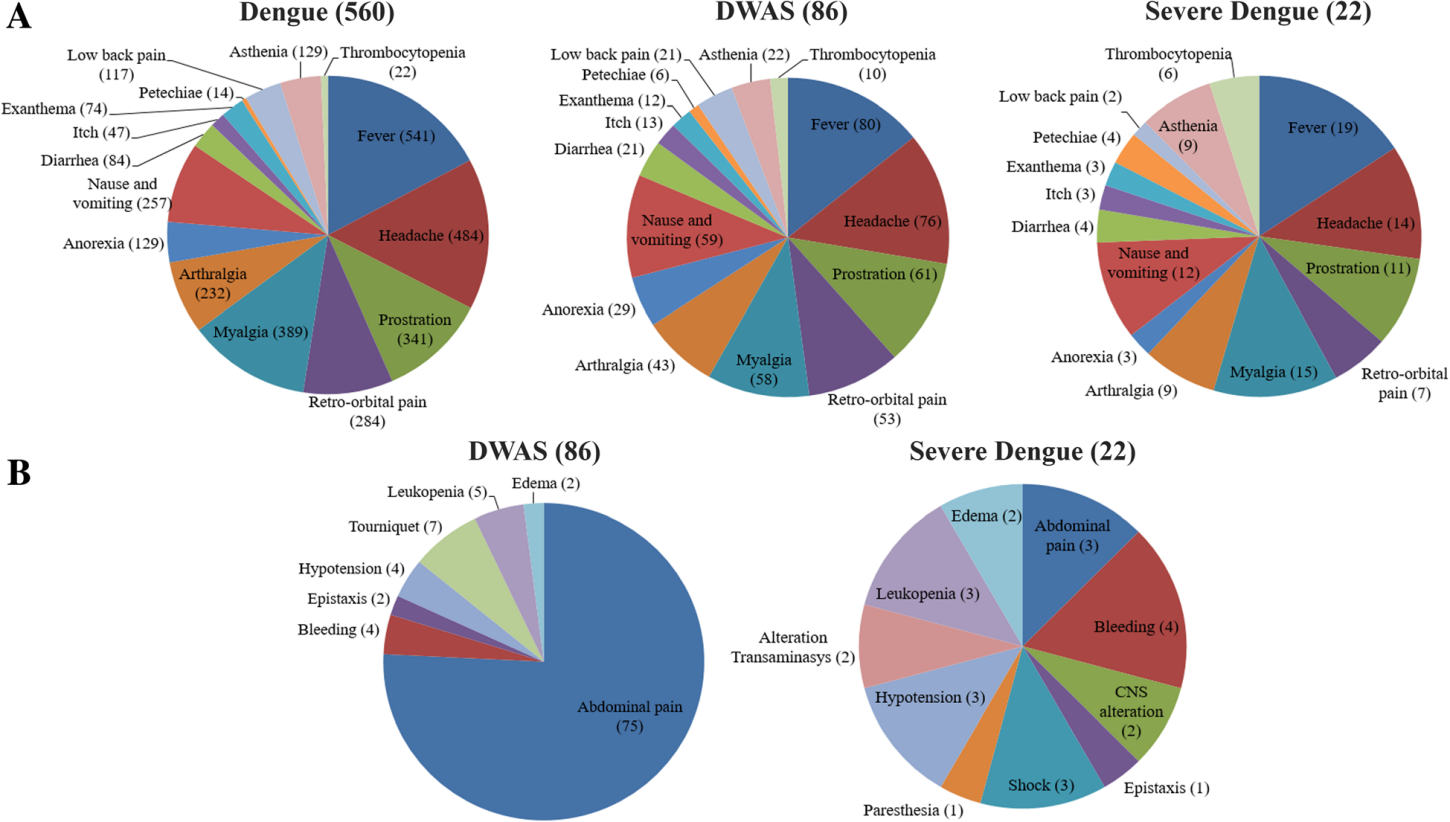
Distribution of clinical manisfestations from dengue, dengue with warning signs (DWAS) and severe DENV-4 cases occurred in Rio de Janeiro form 2011 to 2013

The patient’s immune response characterization in primary or secondary infections was possible in 508 DENV-4 cases, due to the lack of information in some cases allowing this analysis. Overall, primary cases were more frequently observed (259/508; 51.0%) than secondary ones (249/508; 49.0%), and despite the lower number of cases in 2011, no significant differences were observed, Table 3. DENV-4 cases (*n =* 668) were also characterized according to the disease severity. Most of the cases were classified as dengue cases (*n =* 560; 83.8%), 12.8% (*n =* 86) as DWAS and 3.2% (*n =* 22) as severe dengue according to the 2009 WHO criteria, Fig. 2. The clinical manifestations observed in most cases were fever (*n =* 640; 95.8%), followed by headache (*n =* 574; 85.9%), independent of the disease severity. Among the most common symptoms observed on dengue, no differences were observed in relation to the disease severity (Fig. 3a). When observing the warning symptoms, a higher proportion of abdominal pain in DWAS cases was observed (*n =* 75; 87.2%). Despite this, the proportion of abdominal pain in severe dengue cases was considerably lower (*n =* 3; 13.6%), Fig. 3b.

**Table 3:** Immune response characterization of DENV-4 cases occurred in Rio de Janeiro from 2011 to 2013

Type of infection (%)/Year	2011 (*n =* 11)	2012 (*n =* 397)	2013 (*n =* 100)	Total (%) (*n =* 508)
Primary	3 (27.3)	198 (49.9)	58 (58.0)	259 (51.0)
Secondary	8 (72.7)	199 (50.1)	42 (42.0)	249 (49.0)

**Fig. 4:**
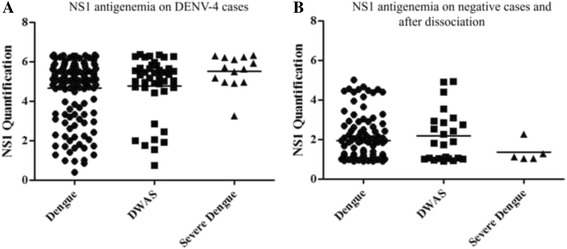
DENV-4 NS1 antigenemia according to the disease severity. **a** NS1 antigenemia on DENV-4 cases positive for NS1 ELISA; (**b**) NS1 antigenemia on DENV-4 cases previoulsy negative by NS1 ELISA and positive after heat-mediated immune-complex dissociation

The NS1 antigenemia on DENV-4 cases (*n =* 138) showed no differences between NS1 levels from dengue, DWAS or severe dengue cases (Fig. 4a). The same was observed on DENV-4 cases previoulsly negative by NS1 ELISA capture and that resulted positive after heat-mediated immune complex dissociation (Fig. 4b). Viremia was also quantified on dengue (*n =* 8) and severe dengue (*n =* 8) cases, however no differences were observed (5.95 log10 RNA copies/mL and 5.83 log10 RNA copies/mL, respectively (*p =* 0,001).

A relationship between the disease severity and the type of infection (primary or secondary) was shown. There was a greater proportion of a more severe disease among individuals 65 years old and older, however a paired analysis by age showed no significant difference in risk of more severe disease (*p > 0.05)* nor for secondary infections (*p > 0.05*), Table 4.

**Table 4:** Analysis of factors associated to DENV-4 cases in Rio de Janeiro, Brazil, from January 2011 to December 2013

	Dengue		*p* value*	DWAS		*p* value*	Severe dengue		*p* value*
	*n*	%		*n*	%		*n*	%	
Age group (years)									
**≤** 15	102	85.0		15	12.5		3	2.5	>0.05
16–64	426	83.8	>0.05	67	13.1	>0.05	15	2.9	
**>** 65	20	83.3		1	4.1		3	12.5	
Type of infection									
Primary	159	48.6	>0.05	43	50.5	>0.05	5	31.2	>0.05
Secondary	168	51.4		42	49.5		11	68.8	

*Statistical analysis performed for the comparison of the disease severity to the distinct age groups and type of infection

Fatal cases due to DENV-4 (*n =* 14) were received and analyzed during the studied period, 42.8% (6/14) occurred in 2012 and 57.1% (8/14) in 2013. Immune response was characterized on 7 cases and, 42.8% (3/7) occurred during a primary infection and 57.1% (4/4), due to secondary ones. Age information was available on 12 cases and, 7 occurred on patients’ 16 to 65 years old, Table 5.

**Table 5:** DENV-4 fatal cases (*n =* 14) occurred from 2011 to 2013 in the State of Rio de Janeiro, Brazil

Year	2012	2013
Type of infection (*n =* 7)	*n*	
Primary (*n =* 3)	2	1
Secondary (*n =* 4)	3	1
Age group (*n =* 12)	*n*	
≤ 15 (*n =* 2)	2	NI
16–65 (*n =* 7)	3	4
> 65 (*n =* 3)	NI	3

*NI* not identified

**Fig. 5:**
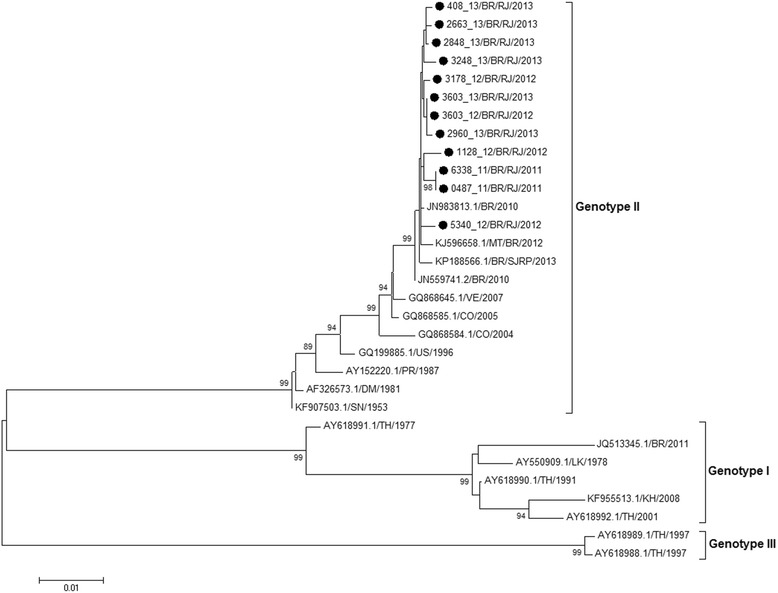
Phylogenetic analysis based on C/prM/M genes from representative DENV-4 (*n =* 12) strains isolated from 2011 to 2013, Rio de Janeiro, Brazil. Neighbor-joining method, Tamura-Nei-parameter model (TN93 + G) with gamma parameter 0,23. Bootstrap test (1000 pseudo-replicates) is presented on the basis of the branches. Black circles represent the DENV-4 sequences analyzed. DENV reference strains were named as follows: number of GenBank accession/country/year

The phylogeny based on the partial genome sequencing of representative DENV-4 strains (*n =* 12) isolated in State of Rio de Janeiro, represented by dengue and one fatal case, demonstrated the circulation of DENV-4 Genotype II (Fig. 5).

## Discussion

The introduction of DENV-4 in the state of Roraima, North Brazil in 2010 warned for the possibility of a new dengue epidemics in Brazil [16], as the main vector, *Aedes aegypti* is widespread in the country. In the disease surveillance, the identification of the vectors has important implications for the disease outbreak control, especially with the rapid disease spread when a new serotype is introduced.

In 2011, the first isolation of DENV- 4 in RJ from cases occurred in the city of Niteroi resulted in the emergence of this serotype in the state in 2012, characterized by isolation of this serotype in 48.7% of confirmed cases. That same year showed for, the first time, the co-circulation of the four DENV serotypes in the state. However, in 2013, DENV-4 was responsible for the largest number of cases in the state.

The Metropolitan region of the state was responsible for most cases occurred during the studied period, mainly in the municipalities of RJ and Niterói. This region has a high population density and high-traffic, which partly explains the concentration of the cases studied [12]. Furthermore, as the Flavivirus Laboratory, FIOCRUZ acts as a Regional Reference Laboratory for dengue diagnosis in Brazil, samples from other municipalities were also received and analyzed.

Although viral isolation is considered the “gold standard” method for dengue viral detection, the sensitivity of the molecular methods in detecting the viral genome offers a great advantage during an epidemic period, as shown by the results presented here. However, it is noteworthy that the high percentage of positive results obtained in RT-PCR may be the result of the selection criteria for the analysis in this study, as known DENV-4 cases were selected. Despite this, previous studies have demonstrated the usefulness of RT-PCR to confirm the cases and identification of the infecting serotype, when the virus isolation was not possible [25, 26].

Serological methods are still the most useful tool for the diagnosis of the disease during epidemics, as observed since its establishment in 1986 in Rio de Janeiro [27]. In this study, the low detection rate of anti-DENV IgM by ELISA (39.4%) was probably due to the acute nature of the cases selected for analysis, as previously described. When it has been described that patients with a primary infection have evidently undetectable IgM antibody titers and there is an increase in detection from a paired sample in the later disease [9]. Usually, higher anti-DENV IgM detection rates are observed after the sixth day after the onset of symptoms. Therefore, considering cases with ≥4 days of symptoms, the detection rate significantly increases.

The usefulness of NS1 Ag capture tests for the early diagnosis of dengue, even in limited resourced setting has been shown [28] and evaluated to confirm primary and secondary acute dengue infections [28–31]. In this study, initially, the NS1 capture ELISA detection rate was 41.5% on previously confirmed DENV-4 cases. However, after using a heat immune complex dissociation step, the NS1 detection rate significantly increased to 87.6%, as previously described.

In this study, hemorrhage, shock, increased transaminases levels and central nervous system involvement were considered for severe cases classification. In the study, 3.2% of DENV-4 confirmed cases were classified as severe dengue and 12.8% as dengue with warning signs. We observed that abdominal pain is an important warning sign regarding the evolution of the disease and which can help in the correct patient management and thus, avoiding increased severity and fatal outcome. However, due to the small number of severe cases available in this study, no statistical analysis on the disease severity was performed. Furthermore, during the study period, no increase in the disease severity was observed and this is in agreement with previous observations in the same period in the state of RJ [32]. The lower severity on DENV-4 cases is known [33] and here, 14 deaths due to DENV-4 were confirmed in the period studied.

No significant differences between the viremia on mild dengue and severe cases were observed, which does not corroborate with previous studies analyzing other serotypes [34–36]. However, a limitation of the present study was the small sample size of severe cases. Moreover, the sampling collection represents the patient’s first visit to the health unit and the information on their progression to a more severe disease is not available.

DENV-4 strains isolated from 2011 to 2013, following this serotype introduction and spread in RJ were partially sequenced and the phylogenetic analysis demonstrated that those belong to genotype II. Moreover, based on previous studies, the sampling size sequenced here is reliable for this analysis considering the epidemic duration, site and the low-mutation characteristic of DENV [16, 37]. The strains of genotype II first isolated during the outbreak of 1982 in Roraima, North Brazil [38] did not spread to other states at that time. However, this genotype was once again detected in Roraima in 2010, being circulated through Central America, Northeastern South America and the Caribbean [39]. According to Temporão et al. (2011) [16], these strains of genotype II were genetically different from those isolated in the 1980s in Roraima and demonstrated a probable origin from Venezuela and Colombia. However, Nunes et al. [40] complete and partial DENV-4 sequences isolated in Brazil identified the circulation of two genotypes (I and II). Genotype II is the most commonly one observed circulating in South America and the Caribbean. Genotype I, one the other hand is represented by a strain isolated in the Northeast of Brazil (Bahia), which may have some evolutionary relationship with Asian strains, according to phylogenetic analysis performed by Campos et al. [41].

## Conclusions

After the DENV-4 isolation in 2011 in RJ, this serotype spread in the following two years and, the co-circulation of the four DENV was evidenced for the first time in the state. The DENV-4 diagnosis by NS1 ELISA was significantly improved by using an immune complex dissociation step. We observed a low percentage of clinical manifestations related to a more severe disease, and despite the occurrence of secondary infections, immune status and age were not related to a more severe disease. Although two distinct DENV-4 genotypes were already reported circulating in Brazil, in this study, genotype II was the only identified during the period in RJ. The impact of the introduction and emergence of DENV-4 in the state of RJ was not well understood and studies accessing a new DENV serotype circulation in an endemic scenario are needed.

## Abbreviations

ALT: Alanine transferase
AST: Aspartate aminotransferase
DENV: Dengue Virus
DHF: Dengue hemorrhagic fever
DwWS: Dengue with warning signs
IFAT: Indirect fluorescent antibody test
Ig: Immunoglobulin
RT-PCR: Reverse transcriptase-polymerase chain reaction
SD: Severe dengue
WHO: World Health Organization

## References

[1] Word Health Organization. Sustaining the drive to overcome the global impact of neglected tropical diseases: second WHO report on neglected tropical diseases. Geneva 2013; 25–29.

[2] Lindenbach B, et al. Flaviviridae: The viruses and their replication. PH KDaH, ed. Fields Virology. 2001; 991–1041.

[3] Lanciotti (1997). Molecular evolution and phylogeny of dengue-4 viruses. Journal of General Virology.

[4] Weaver SC, Vasilakis N (2009). Molecular evolution of dengue viruses: contributions of phylogenetics to understanding the history and epidemiology of the preeminent arboviral disease. Infections, Genetics and Evolution.

[5] Klungthong C, et al. The molecular epidemiology of dengue virus serotype 4 in Bangkok, Thailand. Virology. 2004; 329:168–179. 10.1016/j.virol.2004.08.003 15476884

[6] Claro L (2004). Dengue prevention and control: a review of studies on knowledge, beliefs, and practices. Caderno Saude Publica.

[7] Schatzmayr HG (1986). An outbreak of dengue virus at Rio de Janeiro. Memórias Instituto Oswaldo Cruz..

[8] Nogueira RM (1990). Isolation of dengue virus type 2 in Rio de Janeiro. Memórias Instituto Oswaldo Cruz..

[9] Nogueira RM (1993). Dengue epidemic in the stage of Rio de Janeiro, Brazil, 1990-1: co-circulation of dengue 1 and dengue 2 serotypes. Epidemiol Infect.

[10] Nogueira RM (2001). Dengue virus type 3 in Rio de Janeiro. Brazil Memorias Instituto Oswaldo Cruz.

[11] Nogueira RM (2005). Dengue virus type 3, Brazil, 2002. Emerg Infect dis.

[12] Teixeira M (2008). Recent shift in age pattern of dengue hemorrhagic fever. Brazil. Emerging Infectious Diseases..

[13] Teixeira MG (2009). Dengue: twenty-five years since reemergence in Brazil. Caderno Saude Publica..

[14] Secretaria de Vigilancia em Saude, Ministerio da Saude. Dengue. Boletim da semana 48/2008. In. Brasília: Ministério da Saúde, 2008. http://portalsaude.saude.gov.br/index.php/o-ministerio/principal/secretarias/svs/boletim-epidemiologico. Accessed 13 Dec 2016.

[15] Secretaria de Vigilancia em Saude, Ministerio da Saude. Informe epidemiológico da dengue: Semanas de 1 a 52 de 2009. In Brasília: Ministério da Saúde, 2009 http://Wwwriorjgovbr/web/Sms Accessed 13 Dec 2016.

[16] Temporao JG (2011). Dengue virus serotype 4, Roraima state. Brazil Emerging Infectious Diseases.

[17] Nogueira RM (2011). Dengue virus type 4 arrives in the state of Rio de Janeiro: a challenge for epidemiological surveillance and control. Memorias Instituto Oswaldo Cruz.

[18] Word Health Organization. Dengue: guidelines for diagnosis, treatment, prevention and control. Special Programme for Research and Training in Tropical Diseases. Geneva; 2009. http://www.who.int/tdr/publications/documents/dengue-diagnosis.pdf

[19] Igarashi A (1978). Isolation of a Singh's *Aedes albopictus* cell clone sensitive to dengue and chikungunya viruses. Journal General Virology.

[20] Gubler DJ (1984). Mosquito cell cultures and specific monoclonal antibodies in surveillance for dengue viruses. Am J Trop med Hyg.

[21] Miagostovich MP (1999). Evaluation of an IgG enzyme-linked immunosorbent assay for dengue diagnosis. Journal Clinical Virology.

[22] Lanciotti RS (1992). Rapid detection and typing of dengue viruses from clinical samples by using reverse transcriptase-polymerase chain reaction. Journal Clinical Microbiology.

[23] Johnson BW (2005). Serotype-specific detection of dengue viruses in a fourplex real-time reverse transcriptase PCR assay. Journal Clinical Microbiology..

[24] Lima MRQ (2014). A simple heat dissociation method increases significantly the ELISA detection sensitivity of the nonstructural-1 glycoprotein in patients infected with DENV type-4. J Virol Methods.

[25] Miagostovich MP (1997). Diagnosis of dengue by using reverse transcriptase-polymerase chain reaction. Memórias Instituto Oswaldo Cruz.

[26] Dos Santos FB (2013). A review on dengue diagnosis and epidemiology by a Regional reference laboratory in 25 years, Rio de Janeiro. Brazil Dengue Bulletin.

[27] Andries AC (2012). Field evaluation and impact on clinical management of a rapid diagnostic kit that detects dengue NS1. IgM and IgG PLoS Neglected Tropical Diseases.

[28] Dussart P (2006). Evaluation of an enzyme immunoassay for detection of dengue virus NS1 antigen in human serum. Clin Vaccine Immunol.

[29] Hang VT (2009). Diagnostic accuracy of NS1 ELISA and lateral flow rapid tests for dengue sensitivity, specificity and relationship to viremia and antibody responses. PLoS Negl Trop dis.

[30] Lima Mda R (2010). Comparison of three commercially available dengue NS1 antigen capture assays for acute diagnosis of dengue in Brazil. PLoS Negl Trop dis.

[31] Huang CH, et al. Laboratory diagnostics of dengue fever: An emphasis on the role of commercial dengue virus nonstructural protein 1 antigen rapid test. Journal Microbiology, Immunology and Infection. 2012; doi: S1684–1182(12)00155–7. 10.1016/j.jmii.2012.07.011 23041057

[32] Secretaria de Estado de Saúde e Defesa Civil do Rio de Janeiro (SESDEC-RJ). Relatório de casos de dengue. 2009. http://prefeitura.rio/web/sms/dengue-casos-bairro-periodo. Accessed 14 Apr 2013.

[33] Amâncio FF (2014). Dengue virus serotype 4 in a highly susceptible population in southeast Brazi. Journal Infection Public Health.

[34] Vaughn DW (2000). Dengue viremia titer, antibody response pattern, and virus serotype correlate with disease severity. J Infect dis.

[35] Endy TP (2004). Relationship of preexisting dengue virus (DV) neutralizing antibody levels to viremia and severity of disease in a prospective cohort study of DV infection in Thailand. J Infect dis.

[36] de Araújo JM (2009). Quantification of dengue virus type 3 RNA in fatal and non-fatal cases in Brazil, 2002. Trans R Soc Trop med Hyg.

[37] Barcelos Figueiredo L, et al. Dengue virus 2 American-Asian genotype identified during the 2006/2007 outbreak in Piauí, Brazil reveals a Caribbean route of introduction and dissemination of dengue virus in Brazil. PLoS One. 2014; 9(11). 10.1371/journal.pone.0104516 PMC4134198 25127366

[38] Osanai CH (1983). Dengue outbreak in boa vista, Roraima. Preliminary report. Revista Instituto de Medicina Tropical.

[39] Acosta et al. Dengue virus serotype 4, Roraima State, Brazil. Emerg Infect Dis. 2011; 17:1979–80. 10.3201/eid1710.110776 PMC3310685 22000396

[40] Nunes MR (2012). Phylogeography of dengue virus serotype 4, Brazil, 2010-2011. Emerg Infect dis.

[41] Campos Rde M (2013). Emergence of dengue virus 4 genotypes II b and I in the city of Rio de Janeiro. J Clin Virol.

